# Altered dynamic functional network connectivity in levodopa‐induced dyskinesia of Parkinson's disease

**DOI:** 10.1111/cns.13994

**Published:** 2022-10-13

**Authors:** Qianqian Si, Caiting Gan, Heng Zhang, Xingyue Cao, Huimin Sun, Min Wang, Lina Wang, Yongsheng Yuan, Kezhong Zhang

**Affiliations:** ^1^ Department of Neurology The First Affiliated Hospital of Nanjing Medical University Nanjing China; ^2^ Department of Radiology The First Affiliated Hospital of Nanjing Medical University Nanjing China

**Keywords:** dynamic functional network connectivity, fMRI, levodopa‐induced dyskinesia, Parkinson's disease

## Abstract

**Aims:**

The aim of this study was to clarify the dynamic neural activity of levodopa‐induced dyskinesia (LID) in Parkinson's disease (PD).

**Methods:**

Using dynamic functional network connectivity (dFNC) analysis, we evaluated 41 PD patients with LID (LID group) and 34 PD patients without LID (No‐LID group). Group spatial independent component analysis and sliding‐window approach were employed. Moreover, we applied a k‐means clustering algorithm on windowed functional connectivity (FC) matrices to identify reoccurring FC patterns (i.e., states).

**Results:**

The optimal number of states was determined to be five, the so‐called State 1, 2, 3, 4, and 5. In ON phase, compared with No‐LID group, LID group occurred more frequently and dwelled longer in strongly connected State 1, characterized by strong positive connections between visual network (VIS) and sensorimotor network (SMN). When switching from OFF to ON phase, LID group occurred less frequently in State 3 and State 4. Meanwhile, LID group dwelled longer in State 2 and shorter in State 3. No‐LID group occurred more frequently in State 5 and less frequently in State 3. Additionally, correlation analysis demonstrated that dyskinesia's severity was associated with frequency of occurrence and dwell time in State 2, dominated by inferior frontal cortex in cognitive executive network (CEN).

**Conclusion:**

Using dFNC analysis, we found that dyskinesia may be related to the dysfunctional inhibition of CEN on motor loops and excessive excitation of VIS and SMN, which provided evidence of the changes in brain dynamics associated with the occurrence of dyskinesia.

## Graphical abstract

Using dFNC analysis, we found that dyskinesia may be related to the dysfunctional inhibition of cognitive executive network on motor loops and excessive excitation of visual network and sensorimotor network, which provided evidence of the changes in brain dynamics associated with the occurrence of dyskinesia.

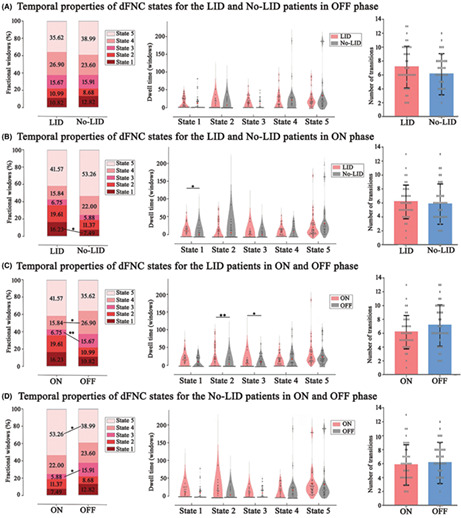

## 1 INTRODUCTION

In Parkinson's disease (PD), levodopa treatments could alleviate motor symptoms. But after 4–6 years, levodopa‐induced dyskinesia (LID), a disabling motor complication, can be induced in 40% of PD patients.^1^, ^2^ Idiopathic PD is considered as a disorder of response initiation characterized by excessive motor inhibition (i.e., akinesia and bradykinesia), while LID is primarily a clinical manifestation of disinhibition of movements.^3^ Previous neuroimaging studies on LID have discovered cortical morphological and functional alterations in inferior frontal cortex (IFC), one fundamental component of executive control or motor inhibition network.^3^, ^4^, ^5^, ^6^, ^7^, ^8^ However, the neural mechanisms behind this motor disorder remain poorly understood.

Traditional static functional connectivity (FC) on LID was measured with the assumption that intrinsic fluctuations during magnetic resonance imaging (MRI) scan were stationary.^3^ Nevertheless, recent studies have proposed that FC is dynamic or fluctuating within seconds to minutes, which highlights the need for detailed inspection of FC along discrete time windows.^9^, ^10^, ^11^ Emerging data suggested the utility of dynamic functional network connectivity (dFNC) with sliding‐window analysis for understanding functional neurodevelopment as well as PD pathogenesis.^12^, ^13^, ^14^, ^15^, ^16^, ^17^, ^18^ Time‐varying FC may reflect implied spontaneous changes in underlying networks, which might improve our knowledge of how neural systems flexibly coordinate to support cognitive and behavioral function. In addition, the interactions between large‐scale brain networks in LID have not been examined in previous studies. The exploration for dynamic internetwork connectivity can provide insights into the abnormal functional integration and specialization of the brain in LID of PD.

Therefore, with dFNC analysis, this study was conducted to investigate the possible neural mechanisms behind LID. To be specific, we explored how large‐scale functional network interactions changed dynamically in temporal domains (fractional windows, dwell time, and number of transitions) of PD patients with and without LID when levodopa worked or did not work. We hypothesized that compared with those without LID, PD patients with LID would display significantly different temporal properties in some specific brain states and help us understand the neural mechanism of dyskinesia.

## 2 METHODS

### 2.1 Participants

In this study, 90 PD patients were consecutively recruited, of whom 50 were clinically diagnosed as LID (LID group), and 40 were patients without LID (No‐LID group). All participants were corresponding to the United Kingdom Parkinson's Disease Society Brain Bank diagnostic criteria for idiopathic PD.^19^ Inclusion criteria were as follows: (1) a minimum 6 months duration of levodopa therapy; (2) presence or absence of LID after an acute levodopa test observed by the neurologist at the last examination; (3) stable levodopa medications dose for 1 month; (4) right handedness; (5) no use of anxiolytic, antidepressant, or antipsychotic drugs; (6) no evidence of severe cognitive impairment, especially dementia [mini‐mental state examination (MMSE) score > 24]; (7) no contraindications for MRI; (8) no evidence of brain tumor, vascular brain lesions, or brain atrophy; (9) no excessive head movements during the MRI scan (see below); and (10) the ability to tolerate 12 h withdrawal of dopaminergic medications before MRI session.

In LID group, all patients presented with peak‐dose dyskinesia rather than diphasic dyskinesia or off‐period dyskinesia. All participants gave their written informed consents. The research was approved by the ethics committee of the First Affiliated Hospital of Nanjing Medical University and completed in line with the Declaration of Helsinki.

### 2.2 Neuropsychological assessment

Clinical and neuropsychological assessments were performed in OFF and ON phase. Hoehn and Yahr (H‐Y) staging scale and Unified Parkinson's Disease Rating Scale III (UPDRS III) were used to evaluate the severity of motor symptoms. The MMSE was adopted to assess cognitive function. Total levodopa equivalent daily dose (LEDD) was calculated for each patient. Besides, Abnormal Involuntary Movement Scale (AIMS) was used to evaluate the severity of abnormal involuntary movements in LID group.^20^ The complete demographic characteristics are listed in Table 1.

**TABLE 1:** Demographics and clinical characteristics of PD patients with and without LID

	LID	No‐LID	*p* Value
*N*	41	34	‐
Age (years)	61.83 ± 9.29	60.53 ± 7.99	0.523^a^
Gender (M/F)	22/19	21/13	0.494^b^
Age at onset (years)	53.00 ± 9.55	55.50 ± 8.08	0.230^a^
MMSE	27.93 ± 1.69	28.21 ± 1.39	0.542^c^
Disease duration(years)	8.29 ± 4.12	5.74 ± 2.94	**0**.**002** ^c^ ^,^ *
H‐Y stage (OFF)	2.52 ± 0.51	2.34 ± 0.56	0.088^c^
H‐Y stage (ON)	2.17 ± 0.38	1.99 ± 0.51	0.077^c^
LEDD, mg/day	730.8 ± 285.7	710.1 ± 383.4	0.309^c^
UPDRS III (OFF)	37.07 ± 14.50	31.15 ± 15.86	0.096^a^
UPDRS III (ON)	21.27 ± 9.27	17.41 ± 12.84	0.136^a^
AIMS (ON)	9.12 ± 6.39	‐	‐

*Note*: Data are given as mean ± standard deviation.

Abbreviations: AIMS, Abnormal Involuntary Movement Scale; F, female; H‐Y stage, Hoehn and Yahr staging; LEDD, Levodopa equivalent daily dose; LID, levodopa‐induced dyskinesia; M, male; MMSE, mini‐mental state examination; *N*, number; No‐LID, without levodopa‐induced dyskinesia; PD, Parkinson's disease; UPDRS, Unified Parkinson's Disease Rating Scale.

^a^ Two‐sample *t*‐test.

^b^ Chi‐squared test.

^c^ Mann–Whitney test.

* *p* < 0.05.

### 2.3 MRI data acquisition

Images were obtained on a 3.0 T Siemens MRI system (Siemens Medical Solutions). Structural 3D T1‐weighted high‐resolution images were obtained using the following sequence (repetition time (TR) = 1900 ms, echo time (TE) = 2.95 ms, flip angle (FA) = 9°, acquisition matrix = 256 × 256, field of view (FOV) = 230 × 230 mm^2^, thickness = 1 mm, gap = 0 mm). Blood oxygenation level‐dependent functional images were acquired with the following parameters: TR = 2000 ms, TE = 21 ms, FA = 90°, in‐plane matrix = 64 × 64, FOV = 256 × 256 mm^2^, thickness = 3 mm, gap = 0 mm, number of slices = 35, number of total volumes = 240.

The resting‐state fMRI ran lasted about 8 min. During the MRI scan, tight foam padding was used to minimize head movement and participants were instructed to keep their eyes closed, stay awake, relax minds, and keep their heads still. All subjects were scanned twice in the same morning immediately before (OFF phase: 12 h after last dopaminergic medication) and ~ 60 min after their usual morning levodopa dose (ON phase), in line with the expected time peaks of LID. The approximate time of LID onset was calculated according to patient's personal medication diary within the last week, which helped us to determine the individual time required for switching from OFF to ON phase. This information was then used to decide when to start fMRI acquisition on the day of the experiment, and scans were immediately ceased as long as maximal dopamine release triggered dyskinesia. Patients were consecutively monitored by clinicians inside the scanner room. Consistent with the studies using the same procedure,^3^, ^4^, ^21^ none of patients in LID group achieved dyskinesia during fMRI scan.

### 2.4 Resting‐state functional MRI preprocessing and head‐motion control

Data preprocessing was conducted using the SPM12 toolbox (http://www.fil.ion.ucl.ac.uk/spm/) implemented in MATLAB software (version R2016b, Math Works, Inc.). The first 10 scans were removed to allow for signal equilibration, resulting in 230 volumes. Then data were corrected for slice timing and head movements. The resulting images were normalized to the Montreal Neurological Institute template and resampled to 3 × 3 × 3 mm^3^, and spatially smoothed with a Gaussian kernel of 6 mm full width at half maximum. To minimize potential head‐motion bias, subjects were excluded if their mean framewise displacement values exceeded 0.5 mm, or maximum displacement in translation indexes *x*, *y*, or *z* was higher than 3 mm or in rotation indexes was higher than 3°.^14^, ^22^, ^23^, ^24^

### 2.5 Group independent component analysis

**FIGURE 1:**
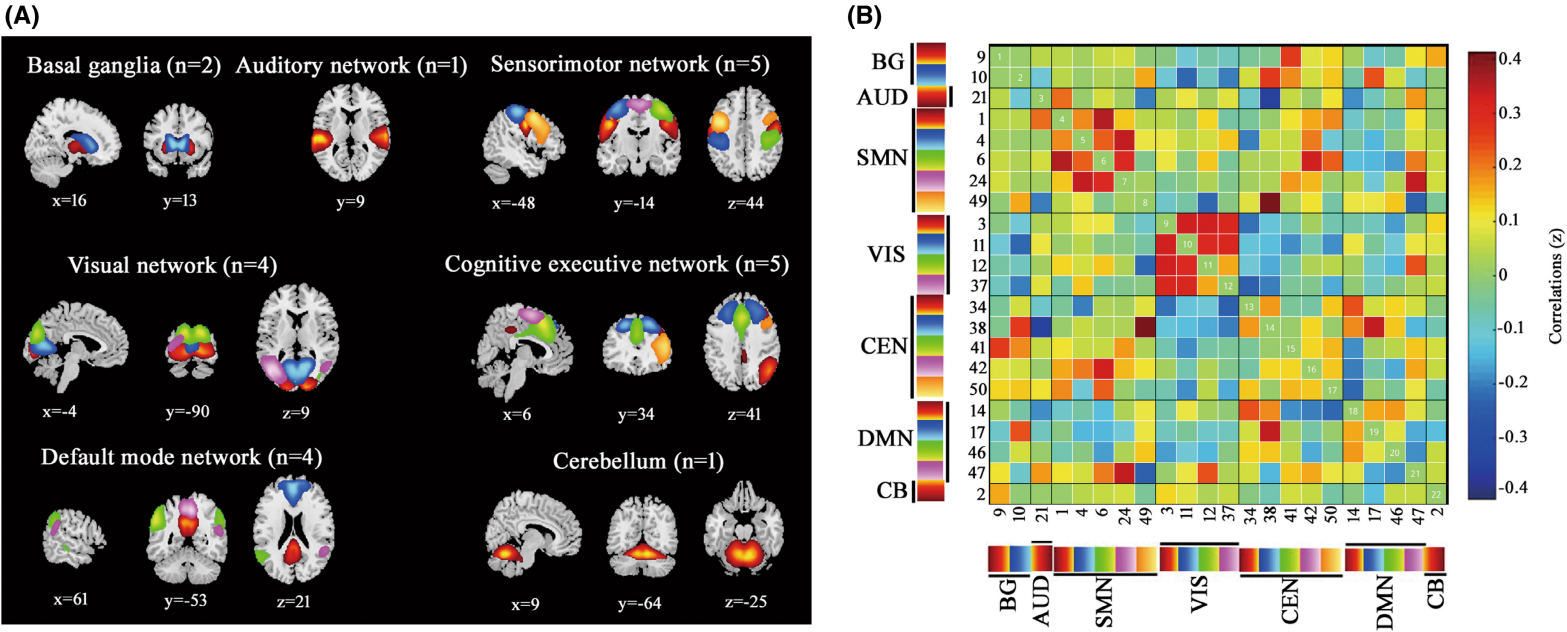
22 independent components identified by group independent component analysis. (A) Seven functional connectivity networks [basal ganglia (BG), auditory network (AUD), sensorimotor network (SMN), visual network (VIS), cognitive executive network (CEN), default mode network (DMN), and cerebellum (CB)] derived from the 22 independent components on the basis of their anatomical and functional properties. (B) Group averaged static functional connectivity between independent component pairs was calculated using the entire resting‐state data. The value in the correlation matrix represented Fisher's z‐transformed Pearson correlation coefficient. Each of the 22 independent components was rearranged by network group based on the seven functional networks.

To create intrinsic networks, we performed spatial group independent component analysis (GICA) implemented in the GIFT toolbox (GIFT v4.0b; http://icatb.sourceforge.net). Subject‐specific principal component analysis was applied to reduce data into 50 independent components (ICs), with the minimum description length criterion.^15^ The stability and validity of ICs was ensured by repeating the ICA algorithm 20 times in ICASSO.^25^, ^26^ Subject‐specific spatial maps and time courses of each IC were created by the GICA back‐reconstruction algorithm.^27^ Of the 50 extracted ICs, relevant intrinsic connectivity networks were identified based on the criteria from Allen.^9^ Firstly, we manually confirmed whether the peak activation clusters of spatial maps were located primarily in gray matter, no spatial overlap with vascular, ventricular, or susceptibility artifacts. Then, we selected ICs with time courses dominated mainly by low‐frequency fluctuations (ratio of power below 0.1 Hz to 0.15–0.25 Hz), and with a high dynamic range in spectrum. This procedure resulted in 22 meaningful ICs, which were sorted into seven networks according to the spatial correlation values between ICs and the resting‐state networks template.^12^, ^14^, ^28^ As shown in Figure 1A and Table 2, the seven networks contained basal ganglia (BG), auditory (AUD), sensorimotor network (SMN), visual (VIS), cognitive executive network (CEN), default mode network (DMN), and cerebellum (CB).

**TABLE 2:** Peak activation information of 22 independent components

Intrinsic connectivity networks	MNI coordinate			
Basal ganglia network (BG)	X	Y	Z	BA
IC 9 R putamen	28	0	0	‐
IC 10 L putamen	−8	12	5	‐
Auditory network (AUD)				
IC 21 L superior temporal lobe	−54	−28	10	22
Sensorimotor network (SMN)				
IC 1 R postcentral gyrus	54	−6	30	4
IC 4 L postcentral gyrus	−36	−26	55	4
IC 6 R precentral gyrus	38	−22	−20	4
IC 24 R paracentral gyrus	6	−30	65	4
IC 49 L precentral gyrus	−42	2	45	6
Visual network (VIS)				
IC 3 R calcarine gyrus	20	−96	0	18
IC 11 R lingual gyrus	12	−62	5	17
IC 12 R superior occipital gyrus	16	−88	30	18
IC 37 L middle occipital gyrus	−42	−66	5	37
Cognitive executive network (CEN)				
IC 34 R inferior parietal lobule	48	−56	50	40
IC 38 L middle frontal gyrus	−18	22	50	44
IC 41 R supplementary motor area	4	18	55	6
IC 42 L supplementary motor area	−14	0	65	6
IC 50 R inferior frontal gyrus	48	24	20	48
Default mode network (DMN)				
IC 14 L precuneus	−4	−66	35	7
IC 17 L medial superior frontal gyrus	0	54	20	9
IC 46 L angular gyrus	−54	−56	35	39
IC 47 R precuneus	6	−56	55	5
Cerebellum network (CB)				
IC 2 cerebellum	8	−64	−25	‐

Abbreviations: BA, Brodmann area; IC, independent component; L, left; MNI, Montreal Neurological Institute; R, right.

The time courses of 22 ICs underwent additional postprocessing to remove remaining noise sources.^15^, ^29^ Time courses were detrended, despiked using 3DDESPIKE algorithm, multiply regressed of the head movement parameters. Then, temporal bandpass filtering with a high‐frequency cutoff of 0.1 Hz was performed. To obtain the static FC matrix, pair‐wise Pearson's correlations were calculated and then transformed to *z*‐values prior to further analysis (Figure 1B).

### 2.6 Dynamic functional connectivity computation

The dFNC analysis was examined by applying two approaches: a sliding‐window approach and k‐means clustering. A sliding‐window approach was adopted to estimate changes of FC over time, and the resulting windowed segments were used to calculate transient FC patterns. In our study, data were segmented into a sliding window of 30 TR,^30^, ^31^, ^32^, ^33^ with a Gaussian window alpha value of 3 TR and a step size between windows of 1 TR. To promote sparsity, a penalty on the L1 norm was imposed utilizing the graphic LASSO framework by 100 repetitions.^34^ To extract reoccurring FC patterns or clusters (also described as states), we applied k‐means clustering algorithm and iterated it 500 times. The similarity between window FC matrix and k‐means cluster centroids was calculated using the L1 distance.^9^ Based on the elbow criterion,^9^, ^17^ the optimal number of cluster centroids (i.e., states) was determined to be five (*k* = 5), the so‐called State 1, 2, 3, 4, and 5. According to the similarity with obtained five cluster centroids, all FC matrices of each subject were classified into one of the five states.

To investigate the temporal properties of dFNC states, we computed three different variables: (1) fractional windows, defined as the proportion of time for total subjects spent in each state, which was calculated by percentage; (2) mean dwell time, representing the time of participants staying in a certain state, as measured by averaging the number of consecutive windows belonging to one state before converting to the other state; and (3) number of transitions, reflected overall number of transitions between states.^12^, ^14^, ^31^

### 2.7 Statistical analyses

Statistical analyses were performed with IBM SPSS statistics 20.0. Normality of data was tested by Kolmogorov–Smirnov method. Two‐sample *t*‐test was adopted for normally distributed variables and non‐parametric test was adopted for non‐normally distributed variables. To be specific, Wilcoxon signed‐rank test was used to compare temporal properties of the two groups from OFF to ON phases, and we applied Mann–Whitney U test to compare the difference in temporal properties between LID group and No‐LID group during ON and OFF phase, respectively. All *p* < 0.05 were considered significant. Then, we performed Spearman's correlation analyses between temporal properties and the severity of dyskinesia (AIMS score) in LID group in ON phase, adjusting for several possible distractions, including age at onset, LEDD, and disease duration [*p* < 0.05, false discovery rate (FDR) correction].^20^, ^35^, ^36^, ^37^

## 3 RESULTS

### 3.1 Demographic and clinical characteristics

We excluded a total of 15 patients, of whom 10 were due to excessive head movements and five were on account of incomplete scans. Eventually, the remaining 41 patients from LID group and 34 patients from No‐LID group were included for further analysis. No significant differences were found in terms of age (*p* = 0.523) and gender (*p* = 0.494) between PD subgroups. However, disease duration was longer in LID group compared with No‐LID group (8.29 ± 4.12 vs. 5.74 ± 2.94, *p* = 0.002). Hence, this factor was included as one of covariates in further correlation analysis. There were no other demographic and clinical differences between the two groups (Table 1).

### 3.2 Intrinsic connectivity networks

Spatial maps of all 22 ICs are shown in Figure 1A. ICs were grouped into seven networks^12^, ^14^: BG (ICs 9 and 10), AUD (IC 21), SMN (ICs 1, 4, 6, 24, and 49), VIS (ICs 3, 11, 12, and 37), CEN (ICs 34, 38, 41, 42, and 50), DMN (ICs 14, 17, 46, and 47), and CB (IC 2). Figure 1B displayed the static FC. The detailed information of ICs is presented in Table 2.

### 3.3 Clustering analysis and dFNC patterns

**FIGURE 2:**
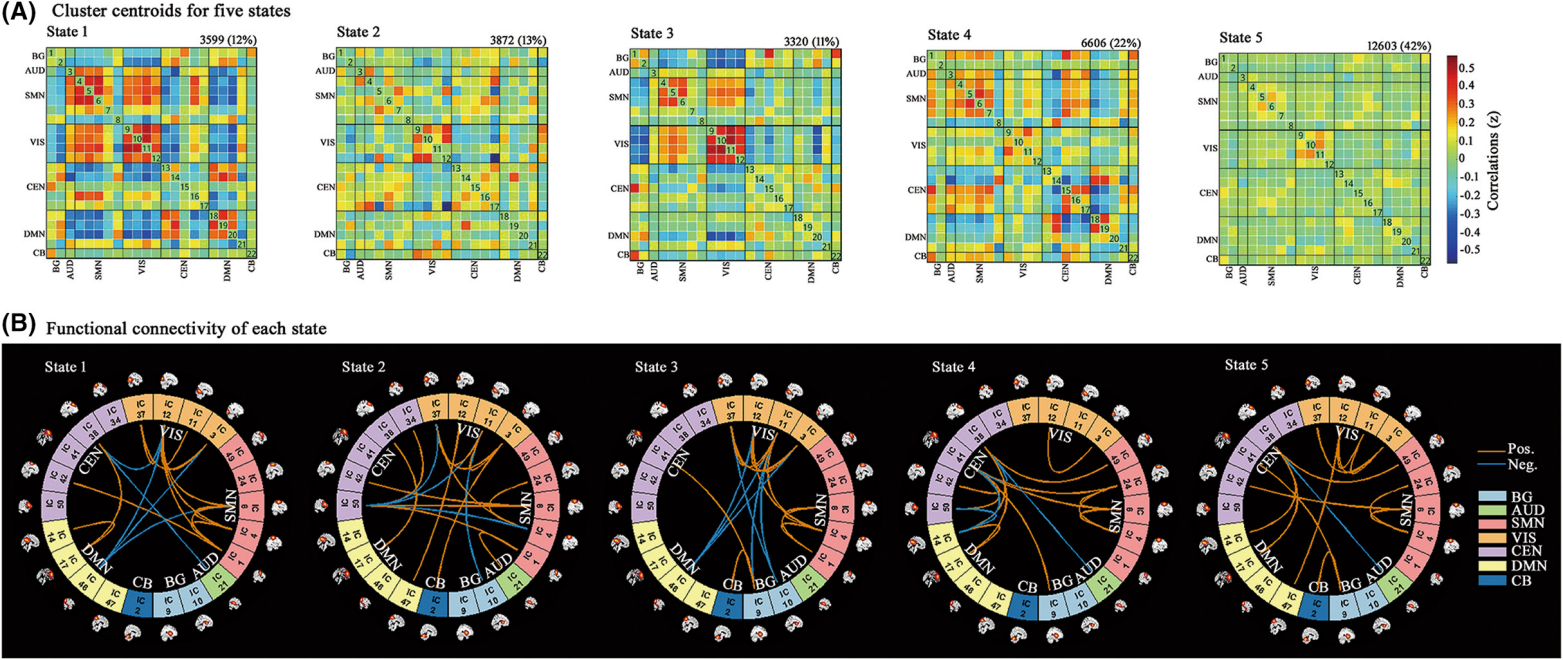
Results of the clustering analysis in each state. (A) Cluster centroids for five states. The total number of occurrences and percentage of total occurrences were listed above each cluster median. (B) Only 5% strongest connections (i.e., the largest absolute value correlation coefficients) in each state was displayed. Orange lines represented positive functional connectivity, and blue lines represented negative functional connectivity. AUD, auditory network;BG , basal ganglia; CB, cerebellum network; CEN, cognitive executive network; DMN, default mode network; SMN, sensorimotor network; VIS, visual network.

Utilizing k‐means clustering, we identified five FC matrices states over the entire MRI scans. As shown in Figure 2A, the percentages of total occurrences of these states were a bit different, with State 5 more frequent (42%) than State 1 (12%), State 2 (13%), State 3 (11%), or State 4 (22%). To better visualize the patterns of outstanding FC differences between states, we kept connections with the most 5% strength of each state (Figure 2B).^14^, ^15^ State 1–5 shared the similarity in within‐network connections of SMN and VIS, while they still possessed some unique between‐network interconnection patterns. State 1 was prominently characterized by strong positive between‐network connections in VIS‐SMN and negative between‐network connections in VIS‐DMN. Centered on CEN (particularly the IC 50), State 2 primarily exhibited strong positive between‐network connections in CEN‐SMN and negative between‐network connections in CEN‐VIS. For State 3, VIS took a vital role, which had strong positive connection with SMN and negative connections with DMN and BG. In State 4, interconnections were centered on CEN (particularly in IC 38 and IC 41), with strong between‐network connections in CEN‐DMN and positive between‐network connections in CEN‐SMN. Combing Figure 2A,B, we found relatively weak within‐network connectivity in SMN and VIS, as well as global neutral between‐network connectivity in State 5.

**FIGURE 3:**
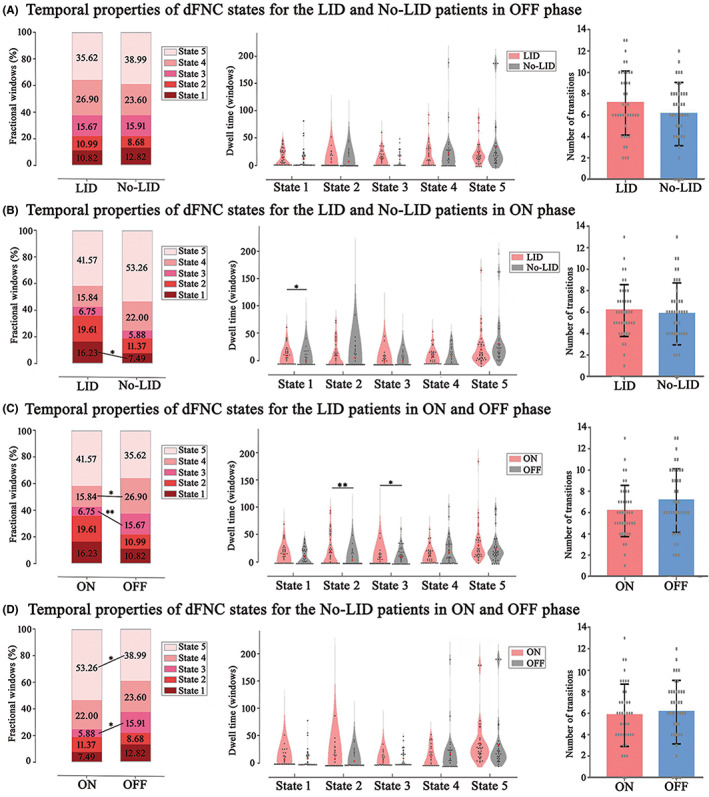
Temporal properties for the PD patients with and without LID, in ON and OFF phases. (A) Temporal properties of dFNC states for the LID and No‐LID patients in OFF phase; (B) Temporal properties of dFNC states for the LID and No‐LID patients in ON phase; (C) Temporal properties of dFNC states for the LID patients in ON and OFF phase; (D) Temporal properties of dFNC states for the No‐LID patients in ON and OFF phase; Red dots in dwell time indicated group medians. *p < 0.05, **p < 0.005. dFNC, dynamic functional network connectivity; LID, levodopa‐induced dyskinesia; No‐LID, without levodopa‐induced dyskinesia; PD, Parkinson's disease

Figure 3 demonstrated temporal properties (fractional windows, mean dwell time, and number of transitions) for each state in OFF and ON phases. In OFF phase (Figure 3A), there were no significant differences between LID and No‐LID group. In ON phase (Figure 3B), compared with No‐LID group, LID group showed increased frequency of occurrence (*p* = 0.042) and dwell time (*p* = 0.039) in State 1 (characterized by strong connections distributed from VIS). When switching from OFF to ON phase, LID group (Figure 3C) occurred less frequently in State 3 (*p* = 0.004) and State 4 (*p* = 0.009). Meanwhile, LID group dwelled longer in State 2 (*p* = 0.002) and shorter in State 3 (*p* = 0.016). These findings suggested that CEN might play an important role in the occurrence of dyskinesia. Meanwhile, when switching from OFF to ON phase, No‐LID group (Figure 3D) occurred more frequently in State 5 (*p* = 0.046) and less frequently in State 3 (*p* = 0.014). Combing these above positive results of the transition from OFF to ON period, we could speculate that levodopa might improve PD symptoms by activating DMN. In terms of number of transitions, we did not find significant differences between groups.

### 3.4 Correlations between dFNC and the severity of dyskinesia in LID in ON phase

**FIGURE 4:**
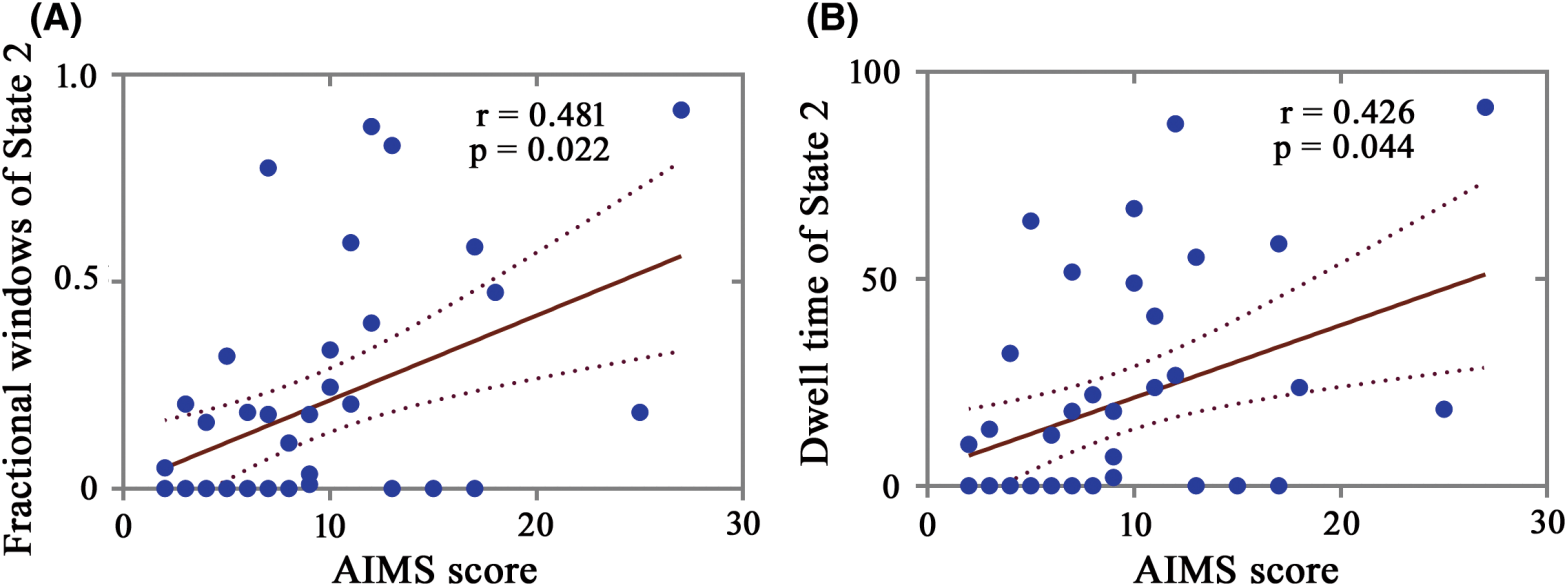
Correlations of AIMS score with temporal properties in the LID group during ON phase. The fractional windows and dwell time of State 2 were positively correlated with the AIMS score in LID group during ON phase (*p* < 0.05, FDR corrected). AIMS, Abnormal Involuntary Movement Scale

After controlling for age at onset, LEDD, and disease duration, we found that fractional windows and dwell time in State 2 of LID group were respectively positively correlated with AIMS score in ON phase (Figure 4; *r* = 0.481, *p* = 0.022; *r* = 0.426, *p* = 0.044; FDR corrected). This indicated that the severer dyskinesia was, the more frequent occurrence and longer time were observed in State 2, dominated by network distributed from CEN. For other states, AIMS score was not significantly correlated with fractional windows and dwell time after FDR corrected. Besides, there were no significant correlations between AIMS score and number of transitions.

## 4 DISCUSSION

In this study, dFNC analysis was used to explore the differences of dynamic functional network connectivity in PD patients with or without LID and on/off levodopa treatments, focusing on temporal properties (fractional windows, dwell time, and number of transitions). Five reoccurring FC patterns or states were identified across the entire sliding time windows. Specifically, LID group occurred more frequently and dwelled longer in State 1 compared with No‐LID group in ON phase. When switching from OFF to ON phase, frequency of occurrence of LID group decreased in State 3 and State 4. Simultaneously, dwell time of State 2 was longer and that of State 3 was shorter. These above results could be interpreted as that after taking medication, the CEN abnormally changed. Functional connectivity between VIS and DMN was more positive than negative. While in No‐LID group, State 5 occurred more frequently and State 3 occurred less frequently when levodopa medications worked. These results reminded us that patients without LID under the influence of drugs displayed increased functional connectivity of DMN. Similar to LID group, the connections between VIS and DMN transformed more from negative connections to positive. Furthermore, correlation analysis indicated that the severity of dyskinesia in LID group was associated with frequency of occurrence and dwell time in State 2.

In this work, we did not focus on definite brain areas of interest as in previous LID studies,^3^ but examined FC of whole brain at the network level. Also, we explored dFNC both in OFF and ON phases, as the occurrence of dyskinesia is closely related to the medicine. Spontaneous brain activity of LID patients, characterized by levodopa medications triggering involuntary movement, is considered as changing over scanning time. The dFNC can capture time fluctuations in network interactions and gain a deeper understanding of basic properties of brain networks.^9^, ^14^ Emerging data suggested the important utility of dFNC for exploring underlying nerve‐damaged mechanisms such as migraine,^17^ epilepsy,^15^ schizophrenia,^13^ and Alzheimer's disease.^16^ These findings implied that dFNC was a promising approach for clinical neuroimaging and might provide greater insights into the neural mechanism of LID.

In this study, no significant differences of temporal properties were found between PD subgroups in OFF phase. In ON phase, compared with No‐LID group, LID group occurred more frequently and dwelled longer in State 1. For State 1, interconnections were found between the VIS and other networks, especially strong positive connectivity between VIS and SMN. This indicated that LID may be related to the excitation of VIS and SMN. VIS is one of the most important sensory perception networks in humans. Since the aberrant processing of visual information has precise influence on motor impairments via sensory guidance,^38^, ^39^ the visual cortex is expected to be excited in LID. Additionally, studies have proved that levodopa partially activates the function of VIS and SMN in PD patients.^40^, ^41^, ^42^, ^43^ However, the exact mechanism of LID needs to be confirmed by further research.

The pathogenesis of LID is still not clear, involving the dopaminergic and serotonergic system.^44^, ^45^ It is believed that aberrant dopaminergic modulation of basal ganglia‐cortical motor loops in the direct and indirect pathways lead to overactivity of frontal cortical areas and the occurrence of peak‐dose LID.^7^, ^46^, ^47^ Our results showed that after taking levodopa, the functional activity of CEN, especially IFC, had been unusually active. In addition, the severity of dyskinesia was only closely linked with frequency of occurrence and dwell time in State 2, dominated by the IFC in CEN, strongly connecting with SMN and VIS. IFC has been included in a critical component of CEN, defined as the motor inhibition network. IFC engages in inhibitory control over motor responses, such as performance monitoring and implementing executive control.^48^, ^49^ Long‐time levodopa treatment may pathologically alter the ability of IFC to monitor motor response and enhance the neural activity in motor cortex either via cortico‐basal ganglia pathway, or via cortico‐cortical pathway.^50^ Our results supported that the dysfunction of IFC and its disinhibition on motor loops (such as VIS and SMN) in response to levodopa could produce an excessive cortical connectivity, and further confirmed the above hypothesis in terms of network level and dynamics of brain activity.^3^, ^4^, ^5^, ^6^ CEN primarily comprising fronto‐parietal regions is involved in key interactive functional networks for coordinating motor function.^51^ It was reported that patients with LID were profoundly relevant to the higher impulsivity score and lower inhibitory control than those without.^52^, ^53^ The SMN is involved in sensory perception and motor process.^54^, ^55^ Studies identified that the disinhibition of cortical–subcortical circuits in the SMN may contribute to the abnormal involuntary movements.^55^ It is conceivable that abnormal interconnections between CEN and SMN, VIS may have impact on inhibition of motor circuits, closely relating to the occurrence of LID.^56^, ^57^ In clinical, it was supposed that modulation of stopping‐related activity in IFC‐subthalamic nucleus (STN) pathway may be therapeutic.^58^, ^59^, ^60^

Both groups under the effect of medicine displayed significantly activation of DMN and the connections between DMN and VIS transformed more from negative to positive. The results suggested that levodopa might improve PD symptoms by altering these connections in somehow. Previous studies have confirmed our speculation. Several literatures described specific malfunctioning of the DMN in cognitively unimpaired PD, probably influenced by the diminished stimulation from striatum.^41^, ^42^, ^43^, ^61^ By comparing functional connectivity of DMN between before and after taking levodopa, Zhong et al.^41^ found that DMN connectivity was indeed impaired, and dopamine was capable of imparting a normalizing effect on DMN, since DMN was regulated by dopamine transmitter. Also, Schneider et al.^40^ found that VIS showed an altered frequency pattern under the effect of levodopa. Both VIS and DMN were speculated to engage in the cognitive management of movement.^62^, ^63^ The influence of levodopa modulated the neural activity in cognitive networks, thus contributed to enhancing movement of PD.^64^

In agreement with previous studies,^9^, ^14^ our results revealed that State 5 was the highest frequency of occurrence, characterized by weak within‐network connections and lack of strong between‐network connections. The frequency of this more common state is thought to be related to the number of self‐focused thoughts,^65^ while other states are considered to reflect cognitive, physiological or motion‐related processes.^66^ When switching from OFF to ON phase, the frequency of occurrence of State 5 increased significantly in patients without LID, correspondingly resulting in the shortage of time spent in other four strongly connected states. Thus, we speculated that when levodopa worked, PD patients without LID might prefer to remain in a calm state, which could be the reason for the absence of LID.

However, a few limitations have to be noted. First, head movements can have a bad effect on resting‐state FC. To alleviate this influence, we performed a series of procedures, but the influence of head movements may not be completely ruled out. Second, similar to previous studies,^37^ the disease duration did not match well in our study, so we have considered this as one of covariates in further analysis to mitigate its effect. Third, to ensure the security of patients and avoid the impact of head movements on image quality, we began fMRI scanning for those patients with LID before levodopa reached its peak in ON phase, which may not fully reflect the functional state of brain during the peak of dose. Last but not least, the data for duration of dyskinesia had not been collected.

## 5 CONCLUSIONS

To summarize, by the dFNC analysis, we found that the presence of dyskinesia may be related to the dysfunctional inhibition of CEN on motor loops and excessive excitation of VIS and SMN. In addition, levodopa may modulate the network connections between DMN and VIS in PD patients. We believe that the results from this study might facilitate our knowledge of understanding the neural mechanism of LID in PD.

## AUTHOR CONTRIBUTIONS

QS, CG, HZ, XC, HS, and LW executed the research project and completed statistical analysis. QS wrote the first draft of the article. MW acquired the data. KZ and YY designed this study and revised the manuscript for intellectual content. All authors approved the final manuscript.

## FUNDING INFORMATION

This study was funded by the National Natural Science Foundation of China (grant numbers 81671258 and 81901297).

## Data Availability

The data that support the findings of this study are available on request from the corresponding author. The data are not publicly available due to privacy or ethical restriction.
